# Content-Based 3D Image Retrieval and a ColBERT-Inspired Re-ranking for Tumor Flagging and Staging

**DOI:** 10.1007/s10278-025-01598-0

**Published:** 2025-08-27

**Authors:** Farnaz Khun Jush, Steffen Vogler, Matthias Lenga

**Affiliations:** Radiology R&D, Bayer AG, Müllerstr. 178, 13353 Berlin, Germany

**Keywords:** Content-based image retrieval (CBIR), Re-ranking, ColBERT, Tumor flagging and staging, Vision embeddings

## Abstract

The increasing volume of medical images poses challenges for radiologists in retrieving relevant cases. Content-based image retrieval (CBIR) systems offer potential for efficient access to similar cases, yet lack standardized evaluation and comprehensive studies. Building on prior studies for tumor characterization via CBIR, this study advances CBIR research for volumetric medical images through three key contributions: (1) a framework eliminating reliance on pre-segmented data and organ-specific datasets, aligning with large and unstructured image archiving systems, i.e., PACS in clinical practice; (2) introduction of C-MIR, a novel volumetric re-ranking method adapting ColBERT’s contextualized late interaction mechanism for 3D medical imaging; and (3) comprehensive evaluation across four tumor sites using three feature extractors and three database configurations. Our evaluations highlight the significant advantages of C-MIR. We demonstrate the successful adaptation of the late interaction principle to volumetric medical images, enabling effective context-aware re-ranking. A key finding is C-MIR’s ability to effectively localize the region of interest, eliminating the need for pre-segmentation of datasets and offering a computationally efficient alternative to systems relying on expensive data enrichment steps. C-MIR demonstrates promising improvements in tumor flagging, achieving improved performance, particularly for colon and lung tumors ($p<0.05$). C-MIR also shows potential for improving tumor staging, warranting further exploration of its capabilities. Ultimately, our work seeks to bridge the gap between advanced retrieval techniques and their practical applications in healthcare, paving the way for improved diagnostic processes.

## Introduction

In the field of computer vision, content-based image retrieval (CBIR) has been extensively studied for decades [1]. Typically, CBIR systems utilize low-dimensional representations (embeddings) of images stored in a database to find similar images based on embedding similarity. Early CBIR methods relied on manually crafted features, which often resulted in loss of important image details due to the constraints of low-dimensional feature design [1–3]. However, recent research in deep learning has focused on generating discriminative feature spaces, resulting in more accurate and efficient CBIR methods [1, 4, 5]. Applying retrieval frameworks to medical images, particularly radiology images, presents ongoing challenges due to the complexity of the task and the nature of medical images, as detailed in [6, 7]. Despite these challenges, the content-based retrieval of medical images offers several advantages, e.g., enabling radiologists to search for reference cases and review historical data, reports, patient diagnoses, and prognoses to enhance their decision-making process [5, 6, 8]. However, in real-world scenarios,medical image data is scarcely annotated and meta-information (such as DICOM headers) is inconsistent or removed, e.g., due to data privacy requirements [9]. This makes manual searching for relevant images extremely time-consuming and impractical for daily clinical routine work [10]. Additionally, progressing research and development in the field of medical imaging requires carefully curated, large datasets. Reliable image retrieval methods can help to further automate data curation, making CBIR an essential tool for supporting future advancements in computer-aided medical image analysis and diagnosis [11].

Moreover, while using advanced feature extraction methods has improved the quality of initial retrievals, refining these results to better match clinical relevance remains critical. Re-ranking techniques−which adjust the order of retrieved items using contextual information, user feedback, or advanced similarity metrics−have emerged as a key strategy to enhance precision in CBIR systems [12, 13]. These methods are particularly valuable in medical imaging, where subtle morphological or pathological differences can impact diagnostic decisions [12, 14].

Previous research has explored the use of hand-crafted feature extraction techniques for CBIR in medical imaging, with a comprehensive review available in [15]. More recent studies have highlighted the potential of pre-trained vision embeddings derived from deep neural networks for CBIR in various applications, including anatomical region retrieval for both 2D [16–18] and 3D images [8, 19, 20], near-duplicate detection in radiology [21], as well as pathological tasks [11, 17, 19, 22]. Notably, the study by [19] introduced the first benchmark utilizing these pre-trained embeddings specifically for tumor flagging and staging. Building on [19], we aim to further investigate and refine the application of CBIR in tumor retrieval, addressing the challenges identified in previous studies and exploring re-ranking strategies to improve the retrieval results.

### Motivation

Integrating CBIR in tumor retrieval is beneficial for enhancing diagnostic accuracy and efficiency in clinical practices. As medical imaging generates vast amounts of data, the ability to swiftly retrieve relevant images based on visual content becomes essential. CBIR systems facilitate this by allowing healthcare professionals to quickly access similar cases, thereby improving the decision-making process. Currently, radiologists often rely on keywords or International Classification of Diseases codes (ICD codes) to locate similar cases within PACS or Radiology Information System (RIS) systems. However, this method has limitations. For instance, the search can be refined significantly if images are included as a condition in the search. Moreover, keyword searches can only retrieve scans that were correctly read and labeled initially, meaning that missed pathologies may not surface in these searches. As such, content-based image similarity search becomes a crucial tool for uncovering missed pathologies from historical records, providing a more comprehensive diagnostic approach. The ability to identify and analyze these missed cases is not only beneficial for patient outcomes but also serves as a valuable feature for quality control departments. The potential to follow up on previously overlooked cases can enhance overall patient care and ensure that health insurance providers are informed of all relevant medical histories. Moreover, the implementation of CBIR can facilitate research and education by providing access to a diverse range of cases, enriching the training of medical professionals, and fostering a deeper understanding of tumor characteristics and variations.

### Prior Work

#### CBIR for Tumor Retrieval

**Fig. 1:**
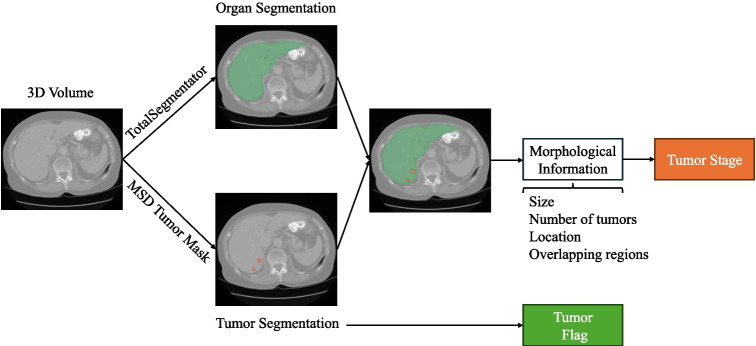
Overview of the 3D image processing to extract tumor information based on [19]: Organ segmentation is performed using the TotalSegmentator model [24]. The tumor segmentation is taken from the MSD ground truth tumor masks provided by [23]. The information from organ segmentation and tumor segmentation is used in combination to extract morphological details, such as size, number of lesions, location, and overlapping regions. This information is then used to derive tumor stages based on the TNM standard [25]

In [19], a CBIR system for tumor flagging and staging is proposed. In their approach, the query consists of an organ that may or may not contain a tumor. Successful retrieval requires accurately matching the tumor status, i.e., whether a tumor is present, and if present, correctly identifying its stage. The experimental setup from [19], relies on data sourced from four tasks of the medical segmentation Decathlon (MSD) challenge dataset [23]. The tumor segmentation is taken from the available ground truth label masks [23]. The organ segmentation is performed using the TotalSegmentator segmentation model [24]. The combined information of organ segmentation and tumor segmentation is used to extract morphological information, e.g., size, number of lesions, location, and overlapping regions. Finally, the tumor stages based on the TNM staging standard [25] are derived. The TNM staging system basically relies on the following parameters: T describes the size of the tumor and any spread of cancer into nearby tissue; N describes the spread of cancer to nearby lymph nodes; and M describes the metastasis (spread of cancer to other parts of the body). The setup proposed by [19] does not include lymph nodes and metastasis due to the unavailability of the related information for the MSD dataset. The staging information based on tumor size (i.e., T) is used to create the train/test or database/query splits and the evaluation of retrieval approaches. The initial setup proposed by [19] is shown in Fig. 1.

The benchmark proposed by [19] relies on two key assumptions that limit its applicability to larger datasets. First, it assumes that **segmentation of each organ is available**, which requires either manual time-consuming delineation or an algorithmic segmentation solution, for example, TotalSegmentator [24]. While AI-based segmentation is a feasible option for many small to medium-sized medical (volumetric) image datasets, it becomes computationally expensive for larger datasets, which are more representative of real-world scenarios where CBIR applications become relevant. Second, [19] created **separate datasets** for each organ. For example, the dataset (and consequently the search space) for colon tumor flagging and staging includes only slices of scans containing the colon. Similar setups are used for the liver and other organs, resulting in four separate datasets for the four organs. While the benchmark in [19] demonstrates the potential of CBIR systems for tumor flagging and staging, its reliance on these assumptions makes it difficult to apply tolarger datasets, thereby limiting a realistic evaluation of the algorithms. Moreover, the presented test scenarios, which assume separated datasets for each organ are not viable in real-world scenarios where data from all organs (or anatomic regions) are stored in the same PACS. Thus, a reality-inspired test set with scans of all relevant anatomical regions would allow a more practical and extended evaluation.

Moreover, the criteria for data splits are not clearly defined. Despite these limitations, [19] provides a valuable starting point for assessing CBIR systems in the context of tumor retrieval and staging. To further enhance the evaluation process, implementing a more generalized dataset and an automatic selection of cases combined with randomization yield a more comprehensive assessment of the algorithms, ultimately improving their applicability in real-world clinical scenarios.

#### Re-ranking

Building upon the foundation of CBIR systems for tumor retrieval, an additional challenge remains in optimizing the relevance of retrieved results. Information retrieval systems aim to provide users with the most relevant results for their queries according to a similarity score. However, initial retrieval results often require refinement to increase the relevance of retrieved information. This refinement process, known as re-ranking, has become an essential component in modern retrieval systems, particularly in CBIR [26]. Re-ranking refers to the process of modifying the order of initially retrieved results to better align with user preferences and requirements. Over the years, numerous approaches have emerged to address this challenge, employing diverse strategies that go beyond pairwise similarity measures [26–30]. One approach is relevance feedback, which involves collecting explicit or implicit input from users about the relevance of specific results. This feedback is then used to adjust the ranking, ensuring that more relevant items appear higher in subsequent searches [27]. Another approach involves learning-based algorithms, which utilize learning-based models to optimize ranking [31–33]. These algorithms analyze features extracted from the data, such as semantic content, visual characteristics, or user interaction patterns, to improve the ranking process. By training models on these features, the system can predict and adjust the relevance of search results, leading to more accurate and personalized retrieval outcomes [28].

More recently, techniques that incorporate contextual information have gained prominence in re-ranking. One such method is ColBERT [13] (Contextualized Late Interaction over BERT [34]). ColBERT addresses the limitations of traditional methods by encoding both documents and queries into rich, multi-vector representations. Instead of relying on single vector embeddings, ColBERT creates an embedding for each token in the query and document. Relevance is then measured by computing the total maximum similarities between each query vector and all vectors within the document. This late interaction architecture allows for a refined and contextually aware retrieval process [13]. Although ColBERT was originally developed for text retrieval, we propose to adopt its contextual late interaction principle for content-based 3D medical image retrieval.

### Contribution

This study expands upon the work of [19] by providing a more comprehensive evaluation of a 3D medical CBIR system on larger, more realistic datasets. We address limitations of prior work by removing the assumptions of pre-existing organ segmentations and organ-specific databases. Additionally, we introduce a novel sampling scheme to construct databases that better represent the true distribution of disease stages. Furthermore, we introduce an innovative re-ranking strategy that considers the 3D image context. The primary contributions of this work are as follows:

- **Organ-Specific Databases with Balanced Stage Distributions:** We propose a systematic sampling method to create four organ-specific databases (colon, liver, lung, pancreas) to ensure balanced representation of different tumor stages.
- **Organ-Agnostic Database for Real-World Applicability:** We developed an organ-agnostic database to better reflect the heterogeneous nature of clinical PACS systems that allows for more realistic evaluation and deployment of CBIR systems.
- **ColBERT Adaptation for Volumetric CBIR and Seg-mentation-Free Retrieval:** We propose a novel adaptation of the ColBERT late interaction method, originally developed for text retrieval [13], for volumetric CBIR that enables context-aware re-ranking of 3D medical images, and eliminates the requirement for pre-existing segmentations by implicitly localizing relevant Regions of Interest (ROIs).
- **Comprehensive Quantitative Evaluation with Statistical Validation:** We conducted a comprehensive quantitative evaluation of our approach across four distinct tumor sites, three feature extractors, and two re-ranking methods.

## Material and Methods

### Vector Database and Indexing

In CBIR, search involves comparing query images against a database of image representations, also referred to as embeddings, to find similarities. In this study, we use cosine similarity to compare embeddings of query images (containing a tumor or not) without using metadata of any kind. Indexing refers to establishing a structure for the efficient storage and retrieval of embeddings. Based on the findings in [8, 35], we selected Hierarchical Navigable Small World (HNSW) [36] as index. The Facebook AI Similarity Search (FAISS) package is used for implementation, specifically, the HNSWFlat index [37]. The overall process can be summarized as extracting embeddings from slices of volumetric images and storing them in a searchable vector database for efficient similarity-based retrieval.

### Feature Extractors

We used three pretrained models as feature extractors, selected to represent a diverse range of training strategies and architectural approaches. Specifically, we included a model that leverages an ensemble of self-supervised and contrastively trained components trained on natural images (DreamSim trained on ImageNet [38]), a model trained with supervised learning on a large medical image dataset (SwinTransformer [39] on RadImageNet [40]), and a model trained with contrastive learning using paired medical images and text (BioMedClip [41]). Previous studies have demonstrated the efficacy of pretrained self-supervised models based on the DINO framework [42–44] for medical retrieval tasks [17, 35]. In this work, we used DreamSim [44] as a representative of this class, specifically we used the ensemble version of DreamSim that consists of DINO model plus CLIP [45] and OpenCLIP [46] and therefore includes strengths of both self-supervised visual representation learning (DINO) and contrastive image-text learning (CLIP/OpenCLIP). Additionally, a SwinTransformer [39] trained on RadImageNet [40] is included based on its reported competitive performance compared to self-supervised models in medical image retrieval [8, 35], offering a strong baseline trained directly on a large-scale medical dataset. Furthermore, the BioMedClip model, previously used for tumor retrieval in [19], was incorporated to provide a point of comparison to existing work in the field and to assess the transferability of a model trained with multi-modal (image and text) data. While fine-tuning pre-trained models on task-specific data can potentially yield further performance gains, we focused on evaluating the zero-shot transfer capabilities of these pre-trained models in this study. This allows us to assess their inherent ability to extract relevant features without task-specific or modality-specific model adaptations. The choice to exclude convolution-based models in this study was mainly motivated by recent advancements of transformer-based models and the DINO framework. It should be noted that this study does not aim at exhaustively benchmarking all available image embedding models in the context of 3D CBIR. Still, we believe that the selected models are diverse enough to provide valuable insights on the capabilities of modern vision embeddings in this context.

**Table 1:** Composition of the MSD challenge dataset, showing number of volumetric scans and axial slices per defined tasks; task 3: colon tumor segmentation, task 6: liver tumor segmentation, task 7: lung tumor segmentation, and task 10: pancreas tumor segmentation

MSD Tasks	3D Volumes	Slices
Task 3	126	13,360
Task 6	131	58,507
Task 7	63	17,594
Task 10	281	26,438
Total	601	115,899

**Table 2:** Overview of number of volumes (Vol.) and embeddings (Emb.) for organ-specific query and database

Organ	Query Vol.	Query Emb.	Database Vol.	Database Emb.	Database Emb.
	(P.+N.)	(P.+N.)	(P.+N.)	w. Segmentation	wo. Segmentation
Colon	60	$5618 \pm 562$	535	$6647 \pm 562$	104,731 ± 1283
Liver	62	$6373 \pm 344$	533	40,614 ± 344	97,802 ± 1247
Lung	28	$4119 \pm 445$	565	43,202 ± 445	108,709 ± 1042
Pancreas	138	$5378 \pm 326$	451	18,016 ± 324	88,879 ± 1865

The notation $P.+N.$ indicates the inclusion of both positive and negative cases. The query set remains consistent across database configurations with (w.) and without (wo.) segmentation. The symbol ± shows the standard deviation of slice counts across 10 experiments using 10 seeds

### Dataset

Following [19], we utilized publicly available data from the MSD challenge [23], specifically, the data from task 3 (colon tumor segmentation), task 6 (liver tumor segmentation), task 7 (lung tumor segmentation) and task 10 (pancreas tumor segmentation). The volumes chosen for the query set and database originate from the MSD training set. The aggregated dataset contains overall 601 3D volumes with 115,899 2D slices, as detailed in Table 1. This data is utilized in the construction of the query and database sets for our experiments. Tumor segmentation masks are taken from the MSD ground truth masks [23] and organ segmentation masks were created for all the 3D volumes utilizing the TotalSegmentator model [24] to facilitate comprehensive comparisons. Figure 1 provides an overview of this process.

**Fig. 2:**
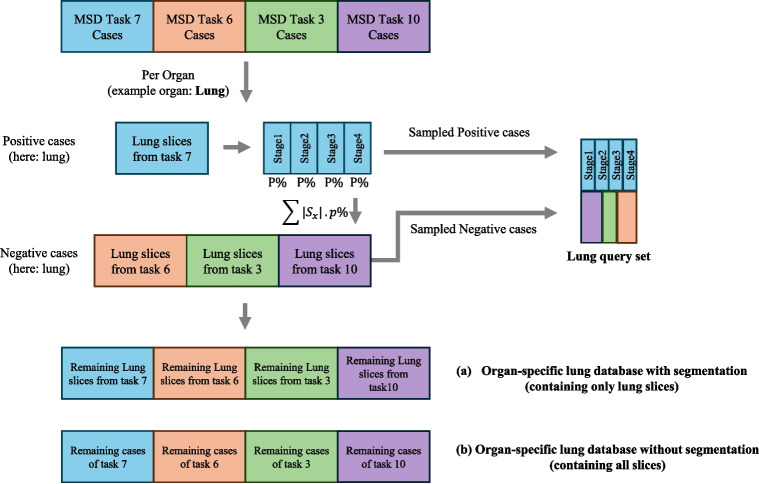
An overview of creating the organ-specific query and databases. For each organ, separate database and query sets are created. In (a), the segmentation masks are used to filter the slices containing lung, which limits the search space to specific lung regions. In contrast, (b) includes all slices in the search space

### Query Setup

We create two different query datasets for our experiment: an organ-specific setup and organ-agnostic setup.

#### Organ-Specific

The organ-specific query set combines tumor-positive and tumor-negative cases, sampled across the selected MSD tasks. For each organ (e.g., lung from Task 7), we created positive and negative query sets. Positive cases were defined as $p\%$ (here 25%) of tumor-containing cases per stage ($S_1,.., S_4$), resulting in $T_p = \sum _{x=1,..,4} |S_x| \cdot p$ total positive cases. Negative cases were matched to the number of positive cases, and consisted of non-tumor slices of the same organ, but taken from other tasks (e.g., slices that contain lung from Tasks 3, 6, and 10 scans). We repeated this sampling process 10 times with different random seeds (sampling is performed with replacement), generating distinct query/database splits for statistical reliability. Embedding counts and case distributions are detailed in Table 2 (Query Vol. and Query Emb. columns). To address the potential correlation between slices within a single 3D volume, we ensured that all slices from a given volume were kept together within the same query/database split. This was achieved by splitting the data at the volume level, rather than the slice level. Figure 2 visualizes the lung-specific query dataset generation as an example.

#### Organ-Agnostic

**Fig. 3:**
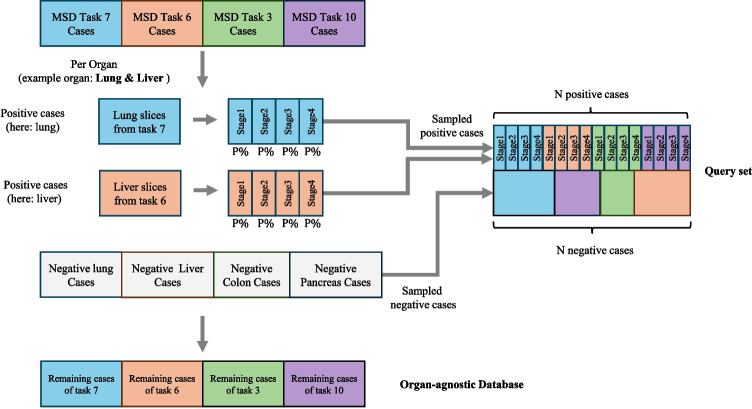
An overview of creating the organ-agnostic query set and database. The query sets and the database encompass all organs and tumor stages. Negative cases are also present in both the query set and the database

The organ-agnostic query set was created by including cases from all four organs (MSD tasks 3, 6, 7, and 10). For each organ, we sampled $p\%$ (here $25\%$ of the tumor-containing cases as positive cases and sampled an equal number of non-tumor cases to maintain a balanced query set. Figure 3 provides an overview of the data generation and Table 3 (Query Vol. and Query Emb. columns) shows the detailed number of cases and slices. The organ-agnostic set was also created by splitting the data at the volume level, ensuring that all slices from a single volume were included in either the query set or the database.

**Table 3:** Overview of number of volumes (Vol.) and embeddings (Emb.) for organ-agnostic query and database

Organ	Query Vol.	Query Emb.	Database Vol.	Database Emb.
	(P.+N.)	(P.+N.)	(P.+N.)	(P.+N.)
All organs	$244 \pm 4$	19,920 ± 761	$356 \pm 4$	65,377 ± 2224

The notation $P.+N.$ indicates the inclusion of both positive and negative cases. The symbol ± shows the standard deviation of slice counts across 10 experiments using 10 seeds

### Database Setup

Our experiments comprise the following different database setups: organ-specific with segmentation, organ-specific without segmentation, and organ-agnostic.

#### Organ-Specific with Segmentation

We created four separate databases, each containing positive and negative cases. After forming the query set (as detailed in Section“Organ-Specific”), the remaining $75\%$ of cases constituted the database. For example, as illustrated in Fig. 2(a) for the lung, the search space is restricted to only the lung slices (with or without tumor). Details of the number of cases and embeddings can be found in Table 2 (“Database Vol.” and “Database Emb. w. Segmentation”columns).

#### Organ-Specific Without Segmentation

Here, we used the same cases as in the “with segmentation” approach (Section“Organ-Specific with Segmentation”) and removed the assumption that organ segmentation masks are available. in Fig. 2(b). Details of the number of cases and embeddings can be found in Table 2 (“Database Vol.” and “Database Emb. wo. Segmentation”columns). As these databases encompass all the slices, they are 1.5 to 4.5 times larger than the databases described in Section“Organ-Specific with Segmentation.”

#### Organ-Agnostic

To simulate a more realistic scenario where all data is stored in a single database, we created an organ-agnostic database by combining images from all tasks, as the example shown in Fig. 3. After establishing the query set (described in Section“Organ-Agnostic”), the remaining cases are stored in a single, unified database. Here, we do not make use of any information derived from image segmentation masks. Table 3 shows the detailed number of cases and slices (“Database Vol.” and “Database Emb.” columns).

### Search and Retrieval

The search is conducted by comparing the similarity of embeddings obtained from the slices of the image volumes. The most straightforward retrieval method involves retrieving for a 2D query slice *q* with the most similar 2D slice $s^*$ from the database. This is done by identifying the slice embedding that maximizes the cosine similarity with the embedding linked to *q*:

1 $$\begin{aligned} s^* = \underset{s \in \text {Database}}{\textrm{argmax}} \frac{\langle \phi (s),\phi (q)\rangle }{\Vert \phi (s) \Vert _2 \Vert \phi (q) \Vert _2} = \underset{s \in \text {Database}}{\textrm{argmax}} \left\langle v_s,\frac{\phi (q)}{\Vert \phi (q) \Vert _2}\right\rangle \end{aligned}$$

where $\langle \cdot ,\cdot \rangle$ denotes standard scalar product, $\Vert \cdot \Vert _2$ the euclidean norm, $\phi$ the embedding mapping and $v_s = \phi (s)/\Vert \phi (s) \Vert _2$ the pre-computed, normalized embedding associated to slice *s* stored in the vector database. Given the query volume $V_Q = [q_1,..., q_n]$, the system retrieves the most similar slice $s_i^*$ from the database for each slice $q_i$ in the query volume, $V_Q$, using Eq. 1. The associated volume ID and its similarity score are then recorded in a hit-table. We implement the Count-base aggregation method from [35], which utilizes a hit-table to determine the volume $V_R$ that has the highest number of hits for the given query volume. To ensure comparability with [19], for each slice query, the 20 most similar slices are considered and the top-k similar volumes are retrieved per each query volume. Moreover, based on the hit-table the maximum similarity score (Max-Score) and the total similarity score (Sum-Sim) are calculated, and two additional top-k volume sets [19] are obtained. The computation of Max-Score and Sum-Sim follows [19] equation 2 and 3, respectively.

**Fig. 4:**
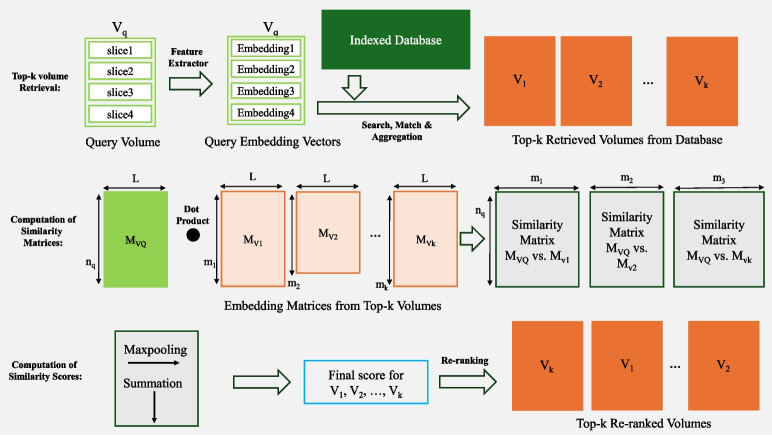
Overview of C-MIR. Image slices vector embeddings are created as explained in Section “Feature Extractors.” The top-k volumes are retrieved based on aggregation criteria presented in Section “Search and Retrieval.” The embedding matrices for the query and the top-k retrieved volumes are utilized to calculate similarity matrices. The rows and columns of all similarity matrices are pooled and summed to compute a rank score per volume, see Section “C-MIR: Colbert-In-spired Medical Image Retrieval and Re-ranking.” Finally, the retrieved volumes are re-ranked based on their rank score

### Re-ranking

#### C-MIR: Colbert-Inspired Medical Image Retrieval and Re-ranking

Inspired by ColBERT [13], here we propose a re-ranking method. To create an analogy to the ColBERT method, each slice can be interpreted as a word, and each volume can be interpreted as a passage. Instead of the BERT encoder [34], for the image retrieval task, the pre-trained vision models are used (see Section“Feature Extractors”). A brief overview of the method is shown in Fig. 4. We call this re-ranking method ColBERT-Inspired Medical Image Retrieval and Re-Ranking (C-MIR).

Once the top-k volumes are selected according to the similarity of individual vectors and the aggregation criteria described in Section“Search and Retrieval,” the selected volumes undergo a re-ranking process:

##### Step 1: Constructing the embedding matrix

For any volume $V=[v_1,...,v_n]$ with *n* slices $v_1,...,v_n$ we can compute the embedding matrix $M_V$ of dimension $n \times L$:

2 $$\begin{aligned} M_V = \left[ \frac{\phi (v_1)}{\Vert \phi (v_1)\Vert _2}, ..., \frac{\phi (v_n)}{\Vert \phi (v_n)\Vert _2} \right] \end{aligned}$$

where $\phi$ assigns each slice to a vector of a constant length *L*, known as an embedding vector. Thus, $M_V$ consists of a collection of embedding vectors derived from the slices.

##### Step 2: Embedding matrix similarity

Assuming another volume $W=[w_1,...,w_m]$ of size $m \times L$ we can compute the similarity matrix of size $n\times m$ via:

3 $$\begin{aligned} \textrm{SIM}(M_V, M_W) = \left[ \frac{\langle \phi (v_i),\phi (w_j) \rangle }{\Vert \phi (v_i)\Vert _2 ~ \Vert \phi (w_j)\Vert _2} \right] _{\begin{array}{c} i=1,...,n\\ j=1,...,m \end{array}} \end{aligned}$$

The entry (*i*, *j*) of this matrix contains the cosine-similarity score of the embeddings related to slice *i* of volume *V* and slice *j* of volume *W* (the extracted embeddings undergo $L_2$ normalization in a postprocessing step; consequently, the dot product becomes equivalent to the cosine similarity). Considering $V_Q$ as the query volume, our re-ranking process begins with calculating the embedding matrix $M_{V_Q}$ according to Eq. 2. Next, we determine the embedding matrices $M_{V_1}, \ldots , M_{V_M}$ for each unique retrieved volume $V_1, \ldots , V_M$ from the initial search, with the top $M=20$ considered (refer to Section“Search and Retrieval”). Subsequently, we calculate the similarity matrices $\textrm{SIM}(M_{V_Q}, M_{V_k})$ for $k = 1, \ldots , M$. In the following step, the similarity matrices are assessed by establishing a final rank score, which is used to reorder the volumes.

##### Step 3: Computing final rank scores and re-ranking

To calculate the final rank score for each volume $V_k$, we first apply max-pooling row-wise to the similarity matrix $\textrm{SIM}(M_{V_Q}, M_{V_k})$. This process identifies the slice in $V_k$ that has the highest cosine similarity to a specific slice in $V_Q$. The resulting vector, which has a length of *n*, is then summed up in order to derive the overall maximum slice similarity, which serves as the final rank score (RS). For $k=1,...,M$, we perform:

4 $$\begin{aligned} \textrm{RS}(V_k) = \sum _{i=1}^n \max _{j=1,...,m_k} \textrm{SIM}(M_{V_Q}, M_{V_k})_{i,j} \end{aligned}$$

where ${V_k}_j$ represents the *j*-th slice of volume $V_k$ and $m_k$ indicates the total number of slices in ${V_k}$. *n* is the number of slices in $V_Q$. The top-k volumes are then re-ranked based on their rank scores (RS), meaning that the volume with the highest score is the most relevant volume, and the volume with the lowest score is the least relevant volume in the top-k results, considering the whole volume slices.

#### Reciprocal Rank Fusion

We compare our re-ranking approach against single aggregation modes and Reciprocal Rank Fusion (RRF) re-ranking approach [47, 48]. RRF is a meta-ranking technique that combines multiple retrieval lists obtained from different retrieval methods in order to leverage the complementary strengths of those [49, 50]. In our setup, we use for RRF the three retrieval methods, i.e., Count-base, Sum-Sim, and Max-Score. Hence, given a query $V_Q$ we generate three ranked lists $L_1, L_2, L_3$ each containing the top 20 retrieved volumes for each method, i.e., $L_\ell = [V_{\ell , 1}, V_{\ell , 2},...,V_{\ell , 20}]$. For any volume $V \in L_1 \cup L_2\cup L_3$ the RRF score is then computed as

5 $$\begin{aligned} RRF(V) = \sum _{\ell =1}^{3} \frac{1}{k + \text {rank}(V, L_\ell )} \end{aligned}$$

where $\text {rank}(V, L_\ell )$ denotes *V*’s rank in the list $L_\ell$. In case *V* is not contained in $L_\ell$, the rank is set to $+\infty$, leading to a zero summand in the overall score. The smoothing parameter *k* is set to 60 following [48, 51]. The final re-ranking is based on the RRF scores, i.e., the retrieved volume with the highest RRF score is assigned the highest final rank.

## Results and Evaluation

This section contains a detailed quantitative evaluation of the retrieval results. Additionally, Appendix 1 includes a visual representation of selected retrieval outcomes for four cases, illustrating both failed and successful instances of tumor flagging and staging through CBIR, which serves to provide the reader with a conceptual framework to better contextualize the quantitative results presented in this section. In line with [19], we used two metrics to evaluate the results: Precision at *k* (P@k) and Average precision (AP). Precision at *k* (P@k) is defined as

6 $$\begin{aligned} P@k = \frac{|{ \text {retrieved cases including tumor in top } k }|}{k} \end{aligned}$$

where $k= 3, 5, 10$. When evaluating information retrieval systems, precision and recall offer a general overview based on the top-k set of retrieved information. However, in many applications, the order in which documents are returned is crucial. Average Precision (AP) is a metric designed to capture this aspect, providing a single-value summary of ranking quality. The average precision (AP) metric is defined as:

7 $$\begin{aligned} AP = \sum _{n=1}^{10} ({R_n - R_{n-1}}){P_n} \end{aligned}$$

where $R_n$ and $P_n$ represent the Recall and Precision at the *n*-th position in the ranked list of top 10 retrieved cases [19, 52, 53]. The formula calculates a weighted average of the precisions at each rank, where the weights are the changes in recall between consecutive ranks. This measures how well the system ranks relevant information higher in the list. The results presented in the following sections show the means of the AP metric, computed across 10 repetitions of the entire experiment, each using a different random seed for case sampling.

### Tumor Flagging

#### Organ-Specific Database

Table 4 shows the performance of re-ranking methods, i.e., C-MIR and RRF for tumor flagging in comparison with the three vanilla aggregation methods for four organs, three different feature extractors and the organ-specific databases with and without segmentation. The results of C-MIR are consistent regardless of the use of segmentation masks, showing the capacity of the method to localize the relevant regions effectively. In our evaluation, C-MIR is consistently the best-performing method for colon tumor flagging for all the models. C-MIR enhances the results for BioMedClip and SwinTransformer embeddings in liver tumor flagging for the databases with segmentation, but performance declines when utilizing DreamSim embeddings. C-MIR is the best-performing method for all models in lung tumor flagging. For flagging pancreatic tumors, C-MIR slightly enhances results with BioMedClip but shows reduced performance for DreamSim and SwinTransformer embeddings.

C-MIR achieves the highest AP of 0.807 for colon tumor flagging using DreamSim embeddings. For liver tumors, the highest AP is 0.811 with the Sum-Sim method, utilizing DreamSim embeddings. In lung tumor flagging, C-MIR using DreamSim embeddings stands out with an AP of 0.942. Regarding pancreas tumor flagging, count-base method using DreamSim embeddings lead, achieving an AP of 0.802 without segmentation and 0.797 with segmentation.The reported metrics represent the average values obtained from 10 experiments, each employing a different random seed for case sampling. A statistical analysis related these results is provided in Section“Statistical Analysis.”

**Table 4:** Overview of **tumor flagging** results using **organ-specific databases** with and without segmentations

	Model	Method	With Segmentation				Without Segmentation			
Colon			p@3	p@5	p@10	AP	p@3	p@5	p@10	AP
	BioMedClip	C-MIR	**0**.**660**	**0**.**655**	**0**.**651**	**0**.**723**	**0**.**660**	**0**.**655**	**0**.**651**	**0**.**723**
		RRF	0.617	0.625	0.624	0.693	0.612	0.616	0.616	0.686
		Count-Base	0.635	0.639	0.629	0.703	0.632	0.630	0.619	0.697
		Max-Score	0.612	0.595	0.593	0.677	0.611	0.598	0.591	0.673
		Sum-Sim	0.635	0.634	0.627	0.701	0.629	0.628	0.618	0.695
	DreamSim	C-MIR	**0**.**759**	**0**.**747**	**0**.**728**	**0**.**807**	**0**.**759**	**0**.**747**	**0**.**728**	**0**.**807**
		RRF	0.730	0.705	0.677	0.771	0.738	0.709	0.683	0.777
		Count-Base	0.709	0.704	0.669	0.757	0.714	0.709	0.673	0.760
		Max-Score	0.726	0.707	0.674	0.782	0.742	0.721	0.681	0.792
		Sum-Sim	0.707	0.703	0.667	0.757	0.714	0.706	0.671	0.759
	Swintrans.	C-MIR	**0**.**739**	**0**.**728**	**0**.**715**	**0**.**787**	**0**.**739**	**0**.**728**	**0**.**715**	**0**.**787**
		RRF	0.680	0.669	0.656	0.736	0.668	0.665	0.650	0.725
		Count-Base	0.658	0.652	0.648	0.718	0.649	0.645	0.641	0.711
		Max-Score	0.653	0.640	0.635	0.713	0.652	0.638	0.628	0.714
		Sum-Sim	0.657	0.649	0.647	0.716	0.648	0.643	0.640	0.710
Liver	BioMedClip	C-MIR	**0**.**749**	**0**.**735**	**0**.**709**	**0**.**792**	**0**.**749**	**0**.**735**	0.709	0.792
		RRF	0.738	0.720	0.707	0.789	0.748	**0**.**735**	0.713	**0**.**797**
		Count-Base	0.742	0.725	0.707	0.781	0.740	0.726	0.717	0.790
		Max-Score	0.723	0.710	0.694	0.778	0.731	0.728	0.707	0.782
		Sum-Sim	0.742	0.724	0.707	0.782	0.739	0.725	**0**.**718**	0.790
	DreamSim	C-MIR	0.737	0.727	0.709	0.787	0.737	0.727	0.709	0.787
		RRF	0.749	**0**.**739**	**0**.**712**	0.797	0.756	0.741	0.718	0.802
		Count-Base	0.759	0.736	**0**.**712**	**0**.**807**	**0**.**764**	**0**.**742**	**0**.**719**	0.810
		Max-Score	0.717	0.704	0.701	0.768	0.719	0.708	0.700	0.771
		Sum-Sim	**0**.**760**	0.736	**0**.**712**	**0**.**807**	0.763	**0**.**742**	0.717	**0**.**811**
	Swintrans.	C-MIR	0.722	0.715	0.696	**0**.**784**	0.722	0.715	0.696	0.784
		RRF	**0**.**732**	**0**.**722**	0.698	0.783	**0**.**734**	**0**.**724**	0.698	**0**.**790**
		Count-Base	0.713	0.712	0.700	0.772	0.718	0.713	**0**.**701**	0.781
		Max-Score	0.708	0.684	0.666	0.759	0.710	0.687	0.673	0.758
		Sum-Sim	0.714	0.713	**0**.**703**	0.772	0.718	0.712	**0**.**701**	0.782
Lung	BioMedClip	C-MIR	**0**.**902**	**0**.**905**	**0**.**896**	**0**.**923**	**0**.**902**	**0**.**905**	**0**.**896**	**0**.**923**
		RRF	0.898	0.893	0.888	0.928	0.893	0.889	0.882	0.919
		Count-Base	0.900	0.887	0.884	0.921	0.886	0.880	0.879	0.912
		Max-Score	0.901	0.888	0.885	0.921	0.890	0.885	0.881	0.911
		Sum-Sim	0.900	0.886	0.884	0.921	0.886	0.881	0.879	0.913
	DreamSim	C-MIR	**0**.**932**	**0**.**926**	**0**.**913**	**0**.**942**	**0**.**932**	**0**.**926**	**0**.**913**	**0**.**942**
		RRF	0.916	0.902	0.885	0.929	0.916	0.903	0.885	0.930
		Count-Base	0.917	0.909	0.885	0.936	0.918	0.907	0.884	0.935
		Max-Score	0.899	0.887	0.873	0.910	0.896	0.887	0.874	0.910
		Sum-Sim	0.917	0.911	0.886	0.935	0.919	0.908	0.886	0.935
	Swintrans.	C-MIR	**0**.**900**	**0**.**894**	**0**.**884**	**0**.**918**	**0**.**900**	**0**.**894**	**0**.**884**	**0**.**918**
		RRF	0.893	0.887	0.870	0.911	0.890	0.890	0.867	0.912
		Count-Base	0.889	0.872	0.868	0.905	0.890	0.874	0.865	0.905
		Max-Score	0.881	0.859	0.851	0.899	0.881	0.860	0.853	0.900
		Sum-Sim	0.889	0.873	0.868	0.904	0.889	0.874	0.865	0.904
Pancreas	BioMedClip	C-MIR	**0**.**756**	0.744	**0**.**729**	0.795	**0**.**756**	**0**.**744**	0.729	**0**.**795**
		RRF	0.748	0.741	0.721	0.791	0.746	0.739	0.729	0.791
		Count-Base	0.753	**0**.**745**	0.724	0.798	0.745	0.741	**0**.**731**	**0**.**795**
		Max-Score	0.738	0.722	0.708	0.780	0.723	0.720	0.712	0.775
		Sum-Sim	0.753	**0**.**745**	0.724	**0**.**799**	0.743	0.739	0.730	0.794
	DreamSim	C-MIR	0.746	0.738	0.723	0.795	0.746	0.738	0.723	0.795
		RRF	0.751	0.743	0.722	0.795	0.759	0.745	0.726	0.799
		Count-Base	**0**.**757**	**0**.**747**	**0**.**726**	**0**.**797**	**0**.**764**	0.748	**0**.**727**	**0**.**802**
		Max-Score	0.735	0.728	0.711	0.787	0.737	0.729	0.714	0.788
		Sum-Sim	0.755	0.745	0.724	**0**.**797**	0.763	**0**.**749**	0.725	0.801
	Swintrans.	C-MIR	0.738	0.726	**0**.**709**	0.789	0.738	0.726	**0**.**709**	0.789
		RRF	0.746	0.728	0.705	0.790	**0**.**749**	**0**.**736**	**0**.**709**	**0**.**794**
		Count-Base	**0**.**749**	**0**.**730**	0.704	**0**.**794**	0.746	0.733	**0**.**709**	0.791
		Max-Score	0.731	0.717	0.692	0.779	0.732	0.718	0.694	0.783
		Sum-Sim	0.748	0.729	0.705	0.793	0.747	0.733	**0**.**709**	0.791

Reported metrics represent the average values across 10 experiments, each employing a different random seed for case sampling. The bold-faced value in each sub-column shows the best method for each model

#### Organ-Agnostic Database

Table 5 shows the performance of re-ranking methods, i.e. C-MIR and RRF in comparison with the three vanilla aggregation methods for four organs, three feature extractors, and for the organ-agnostic database. C-MIR is the best-performing method for colon tumor flagging across all models. For liver tumor flagging both re-ranking methods slightly improve the results for BioMedClip and SwinTransformer embeddings but show a decline for DreamSim embeddings, a pattern observed similarly in organ-specific databases. C-MIR is the best-performing method for lung tumor flagging for DreamSim embeddings but shows similar performance for other embeddings. RRF outperforms C-MIR using BioMedClip embeddings. For pancreas tumor flagging C-MIR improves the results for all the models and outperforms RRF.

The highest AP for colon tumor flagging is 0.761 using C-MIR with DreamSim embeddings. For liver tumor flagging, RRF and C-MIR perform on par with an AP of 0.79 using BioMedClip embeddings. The best-performing method for lung tumor flagging is count-base and C-MIR method using SwinTransformer embeddings with an AP of 0.88. For pancreas tumor flagging, the highest AP belongs to the C-MIR using DreamSim embeddings with AP of 0.867. Expanding the database allows us to observe the effect of embedding selection on individual tasks. Given the correct choice of embedding for the organ-agnostic database, C-MIR shows a promising performance compared to the vanilla aggregation methods and RRF.

**Table 5:** Overview of **tumor flagging and staging** results using **organ-agnostic database**

	Model	Method	Flagging				Stagging			
Colon			p@3	p@5	p@10	AP	p@3	p@5	p@10	AP
	BioMedClip	C-MIR	**0**.**621**	**0**.**614**	**0**.**606**	**0**.**685**	0.524	0.518	**0**.**514**	**0**.**584**
		RRF	0.596	0.597	0.590	0.662	0.517	0.513	0.508	0.574
		Count-Base	0.602	0.598	0.593	0.668	**0**.**528**	0.518	0.510	0.582
		Max-Score	0.587	0.586	0.580	0.659	0.505	0.503	0.501	0.559
		Sum-Sim	0.599	0.596	0.595	0.666	0.526	**0**.**519**	**0**.**514**	0.582
	DreamSim	C-MIR	**0**.**706**	**0**.**696**	**0**.**676**	**0**.**761**	**0**.**557**	**0**.**556**	**0**.**541**	**0**.**642**
		RRF	0.675	0.660	0.643	0.731	0.551	0.541	0.530	0.624
		Count-Base	0.660	0.653	0.638	0.718	0.545	0.539	0.528	0.617
		Max-Score	0.680	0.659	0.636	0.736	0.547	0.533	0.520	0.622
		Sum-Sim	0.662	0.656	0.639	0.721	0.547	0.542	0.530	0.620
	Swintrans.	C-MIR	**0**.**681**	**0**.**670**	**0**.**665**	**0**.**738**	**0**.**555**	**0**.**545**	**0**.**542**	**0**.**619**
		RRF	0.637	0.633	0.628	0.698	0.535	0.532	0.527	0.597
		Count-Base	0.614	0.620	0.624	0.684	0.526	0.528	0.526	0.594
		Max-Score	0.625	0.620	0.617	0.696	0.525	0.521	0.515	0.594
		Sum-Sim	0.615	0.621	0.626	0.684	0.530	0.532	0.529	0.594
Liver	BioMedClip	C-MIR	**0**.**747**	**0**.**739**	0.712	0.798	0.600	**0**.**600**	0.589	**0**.**674**
		RRF	0.744	0.734	**0**.**713**	**0**.**799**	0.594	0.596	**0**.**591**	0.673
		Count-Base	0.744	0.732	0.708	**0**.**799**	0.592	0.589	0.582	0.670
		Max-Score	0.739	0.724	0.698	0.786	**0**.**606**	0.599	0.585	0.670
		Sum-Sim	0.735	0.721	0.700	0.792	0.583	0.578	0.574	0.663
	DreamSim	C-MIR	0.593	0.569	0.538	0.671	0.382	0.369	0.358	0.487
		RRF	0.620	0.588	0.540	0.692	0.388	0.378	0.359	0.492
		Count-Base	0.632	0.590	**0**.**543**	0.705	0.397	0.376	0.359	0.505
		Max-Score	0.587	0.550	0.521	0.651	0.386	0.367	0.354	0.474
		Sum-Sim	**0**.**635**	**0**.**592**	0.545	**0**.**707**	**0**.**402**	**0**.**379**	**0**.**361**	**0**.**507**
	Swintrans.	C-MIR	**0**.**708**	**0**.**687**	0.663	**0**.**759**	**0**.**559**	0.546	**0**.**533**	**0**.**635**
		RRF	0.706	0.686	0.660	0.759	0.559	**0**.**548**	0.527	0.638
		Count-Base	0.695	**0**.**687**	**0**.**664**	0.754	0.540	0.539	0.526	0.630
		Max-Score	0.667	0.651	0.635	0.731	0.545	0.537	0.525	0.626
		Sum-Sim	0.692	0.685	0.663	0.754	0.538	0.537	0.524	0.630
Lung	BioMedClip	C-MIR	0.829	0.821	0.810	0.857	0.431	0.436	0.444	0.539
		RRF	0.825	**0**.**829**	0.814	**0**.**872**	0.439	0.441	0.442	0.546
		Count-Base	0.832	0.826	**0**.**817**	0.868	0.439	0.436	**0**.**446**	**0**.**550**
		Max-Score	0.832	0.826	0.811	0.857	**0**.**455**	** 0.451**	0.441	0.541
		Sum-Sim	**0**.**834**	0.827	**0**.**817**	0.869	0.440	0.438	**0**.**446**	**0**.**550**
	DreamSim	C-MIR	**0**.**821**	**0**.**800**	**0**.**780**	**0**.**843**	**0**.**513**	**0**.**500**	**0**.**491**	**0**.**588**
		RRF	0.808	0.789	0.749	0.829	0.510	0.497	0.477	0.583
		Count-Base	0.817	0.794	0.757	0.837	0.511	0.494	0.478	0.584
		Max-Score	0.779	0.755	0.724	0.803	0.486	0.483	0.466	0.559
		Sum-Sim	0.819	0.796	0.758	0.838	**0**.**513**	0.496	0.481	0.584
	Swintrans.	C-MIR	0.860	**0**.**857**	**0**.**824**	0.881	0.483	0.481	**0**.**469**	0.577
		RRF	0.856	0.846	0.815	0.879	0.483	0.484	0.469	0.579
		Count-Base	0.864	0.850	0.822	**0**.**882**	0.485	0.483	0.467	**0**.**583**
		Max-Score	0.830	0.813	0.786	0.854	0.474	**0**.**469**	0.453	0.571
		Sum-Sim	**0**.**867**	0.849	0.822	0.881	**0**.**486**	0.484	0.468	**0**.**583**
Pancreas	BioMedClip	C-MIR	**0**.**793**	**0**.**782**	**0**.**765**	**0**.**828**	**0**.**591**	**0**.**583**	**0**.**569**	**0**.**656**
		RRF	0.771	0.769	0.749	0.815	0.575	0.572	0.552	0.645
		Count-Base	0.776	0.768	0.754	0.820	0.584	0.572	0.559	0.648
		Max-Score	0.742	0.739	0.721	0.792	0.544	0.544	0.532	0.624
		Sum-Sim	0.774	0.767	0.752	0.818	0.580	0.569	0.554	0.645
	DreamSim	C-MIR	0.838	**0**.**826**	**0**.**815**	**0**.**867**	0.528	0.520	0.512	**0**.**612**
		RRF	0.825	0.821	0.803	0.861	0.529	0.519	0.502	0.609
		Count-Base	0.833	0.819	0.803	0.860	0.535	0.525	0.507	0.609
		Max-Score	0.812	0.804	0.789	0.847	0.510	0.500	0.484	0.591
		Sum-Sim	**0**.**840**	**0**.**826**	0.810	0.864	**0**.**541**	**0**.**530**	**0**.**513**	**0**.**612**
	Swintrans.	C-MIR	**0**.**777**	**0**.**762**	**0**.**751**	**0**.**815**	**0**.**592**	**0**.**579**	**0**.**566**	**0**.**660**
		RRF	0.766	0.753	0.734	0.806	0.580	0.569	0.553	0.653
		Count-Base	0.761	0.749	0.735	0.805	0.577	0.569	0.557	0.651
		Max-Score	0.725	0.714	0.704	0.778	0.538	0.531	0.522	0.626
		Sum-Sim	0.769	0.756	0.740	0.811	0.584	0.575	0.561	0.656

Reported metrics represent the average values obtained from 10 experiments, each employing a different random seed for case sampling. The bold-faced value in each sub-column shows the best method for each model

### Tumor Staging

**Table 6:** Overview of **tumor staging** results using **organ-specific databases** with and without segmentation

	Model	Method	With Segmentation				Without Segmentation			
Colon			p@3	p@5	p@10	AP	p@3	p@5	p@10	AP
	BioMedClip	C-MIR	0.529	**0**.**529**	**0**.**521**	**0**.**607**	0.529	**0**.**529**	**0**.**521**	**0**.**607**
		RRF	0.523	0.519	0.517	0.594	0.521	0.521	0.517	0.592
		Count-Base	**0**.**534**	0.528	0.519	0.606	**0**.**535**	0.524	0.519	0.604
		Max-Score	0.506	0.499	0.499	0.570	0.508	0.504	0.499	0.572
		Sum-Sim	**0**.**534**	0.528	0.519	0.606	0.533	0.524	0.519	0.602
	DreamSim	C-MIR	**0**.**571**	**0**.**568**	**0**.**556**	**0**.**665**	0.571	**0**.**568**	**0**.**556**	**0**.**665**
		RRF	0.579	0.567	0.541	0.658	0.575	0.565	0.544	0.659
		Count-Base	0.566	0.561	0.539	0.644	0.569	0.565	0.543	0.642
		Max-Score	**0**.**571**	0.554	0.536	0.653	**0**.**578**	0.565	0.543	0.661
		Sum-Sim	0.565	0.561	0.539	0.645	0.569	0.563	0.542	0.642
	Swintrans.	C-MIR	**0**.**568**	**0**.**558**	**0**.**549**	**0**.**648**	**0**.**568**	**0**.**558**	**0**.**549**	**0**.**648**
		RRF	0.551	0.543	0.535	0.62	0.547	0.543	0.534	0.614
		Count-Base	0.550	0.543	0.534	0.612	0.542	0.538	0.532	0.605
		Max-Score	0.526	0.514	0.507	0.593	0.537	0.523	0.509	0.605
		Sum-Sim	0.549	0.543	0.535	0.611	0.542	0.538	0.531	0.604
Liver	BioMedClip	C-MIR	**0**.**608**	0.596	**0**.**589**	0.668	**0**.**608**	0.596	0.589	**0**.**668**
		RRF	0.599	0.585	0.587	0.669	0.602	0.592	0.586	0.664
		Count-Base	0.594	0.583	0.580	0.662	0.585	0.577	0.582	0.658
		Max-Score	0.597	**0**.**598**	0.591	**0**.**670**	0.605	**0**.**601**	**0**.**594**	0.665
		Sum-Sim	0.592	0.582	0.581	0.662	0.584	0.578	0.582	0.657
	DreamSim	C-MIR	0.615	0.608	**0**.**601**	0.677	0.615	0.608	**0**.**601**	0.677
		RRF	0.619	0.613	0.601	0.683	0.619	0.616	0.606	0.686
		Count-Base	**0**.**624**	**0**.**610**	0.599	0.688	**0**.**627**	**0**.**615**	**0**.**607**	**0**.**691**
		Max-Score	0.605	0.600	0.600	0.669	0.608	0.602	0.601	0.669
		Sum-Sim	**0**.**624**	0.609	0.599	**0**.**689**	0.626	**0**.**615**	0.606	**0**.**691**
	Swintrans.	C-MIR	0.599	**0**.**599**	**0**.**585**	**0**.**679**	0.599	0.599	**0**.**585**	**0**.**679**
		RRF	0.608	0.601	0.581	0.683	0.612	0.603	0.583	0.692
		Count-Base	0.589	0.589	0.582	0.669	0.597	0.591	0.579	0.677
		Max-Score	**0**.**605**	0.589	0.578	0.677	**0**.**611**	**0**.**593**	0.584	0.671
		Sum-Sim	0.590	0.589	0.583	0.669	0.596	0.592	0.579	0.678
Lung	BioMedClip	C-MIR	0.662	0.660	**0**.**666**	0.739	0.662	0.660	**0**.**666**	**0**.**739**
		RRF	0.682	0.659	0.66	0.738	0.68	0.657	0.655	0.731
		Count-Base	0.676	0.648	0.653	0.725	0.663	0.645	0.651	0.719
		Max-Score	**0**.**685**	**0**.**666**	0.664	**0**.**741**	**0**.**676**	**0**.**666**	0.660	0.730
		Sum-Sim	0.676	0.647	0.654	0.725	0.663	0.646	0.651	0.720
	DreamSim	C-MIR	**0**.**673**	**0**.**679**	**0**.**670**	0.731	0.673	**0**.**679**	**0**.**670**	0.731
		RRF	0.669	0.662	0.657	0.736	0.667	0.666	0.656	0.738
		Count-Base	0.666	0.661	0.653	0.735	**0**.**670**	0.661	0.654	0.737
		Max-Score	0.639	0.649	0.654	0.705	0.637	0.649	0.656	0.705
		Sum-Sim	0.667	0.663	0.654	**0**.**737**	**0**.**670**	0.661	0.656	**0**.**738**
	Swintrans.	C-MIR	**0**.**669**	**0**.**669**	**0**.**671**	0.727	**0**.**669**	**0**.**669**	**0**.**671**	0.727
		RRF	0.663	0.666	0.663	0.72	0.663	0.676	0.662	0.724
		Count-Base	0.663	0.664	0.659	0.718	0.661	**0**.**669**	0.659	0.722
		Max-Score	0.662	0.654	0.656	**0**.**732**	0.661	0.656	0.657	**0**.**731**
		Sum-Sim	0.664	0.664	0.658	0.718	0.661	**0**.**669**	0.659	0.722
Pancreas	BioMedClip	C-MIR	**0**.**562**	**0**.**554**	**0**.**539**	0.628	**0**.**562**	**0**.**554**	0.539	**0**.**628**
		RRF	0.558	0.549	0.534	0.63	0.552	0.547	0.54	0.625
		Count-Base	**0**.**562**	**0**.**554**	**0**.**539**	**0**.**633**	0.559	0.549	**0**.**542**	0.627
		Max-Score	0.557	0.541	0.526	0.626	0.537	0.537	0.532	0.617
		Sum-Sim	0.560	0.553	0.537	**0**.**633**	0.556	0.545	0.539	0.625
	DreamSim	C-MIR	0.551	0.539	0.534	0.629	0.551	0.539	0.534	0.629
		RRF	0.565	0.552	0.534	0.642	0.575	0.554	0.535	0.644
		Count-Base	**0**.**572**	**0**.**559**	**0**.**540**	**0**.**642**	**0**.**575**	**0**.**562**	**0**.**539**	**0**.**645**
		Max-Score	0.544	0.537	0.521	0.633	0.547	0.536	0.520	0.631
		Sum-Sim	0.570	0.557	0.537	0.641	0.573	0.562	0.536	**0**.**645**
	Swintrans.	C-MIR	0.557	0.547	0.528	0.637	0.557	0.547	0.528	0.637
		RRF	0.564	0.553	0.534	0.641	**0**.**562**	**0**.**555**	0.533	**0**.**641**
		Count-Base	**0**.**572**	**0**.**562**	**0**.**536**	**0**.**645**	0.563	**0**.**555**	**0**.**535**	0.640
		Max-Score	0.554	0.542	0.514	0.630	0.546	0.536	0.515	0.630
		Sum-Sim	0.570	0.560	0.535	0.644	0.562	0.554	0.534	0.639

Reported metrics represent the average values obtained from 10 experiments, each employing a different random seed for case sampling. The bold-faced value in each sub-column shows the best method for each model

#### Organ-Specific Database

Table 6 presents the performance of re-ranking methods, i.e., C-MIR and RRF for tumor staging, in comparison with the three aggregation techniques across four organs and three feature extractors for organ-specific databases, with and without segmentation. C-MIR has the highest performance for colon tumor staging for all the models. For the staging of liver tumors, re-ranking enhances the results of BioMedClip and SwinTransformer embeddings to some extent, yet no clear, consistent trend emerges. On the other hand, DreamSim embeddings demonstrate a decline in performance. For lung tumor staging, C-MIR enhances the results, specifically for BioMedClip and SwinTransformer embeddings, but demonstrates a decrease in performance of DreamSim embeddings. RRF follows a similar trend as C-MIR. In the context of pancreas tumor staging, C-MIR improved the performance of BioMedClip embeddings, although it led to declines for DreamSim and SwinTransformer embeddings. It is noteworthy that the C-MIR results are consistent for both databases, demonstrating its capability to localize relevant regions without requiring prior segmentation to choose organ slices.

C-MIR, employing DreamSim embeddings, achieved the highest AP of 0.665 for colon tumor staging. For liver tumor staging, the highest AP is 0.689 for the Sum-Sim method using DreamSim embeddings for the database with segmentation and 0.691 for the database without segmentation. The best-performing method for lung tumor staging is C-MIR using BioMedClip embeddings with an AP of 0.739 for the database without segmentation and 0.741 using Max-Score for the database with segmentations. For staging pancreatic tumors, the highest AP is achieved by the DreamSim embeddings, using the count-based method with an AP of 0.645 without segmentation, and by the SwinTransformer embeddings, also employing the count-based method, with an AP of 0.645 with segmentation.

#### Organ-Agnostic Database

Table 5 shows the performance of re-ranking methods, i.e., C-MIR and RRF for tumor staging in comparison with the three vanilla aggregation methods for four organs and three feature extractors for the organ-agnostic database. C-MIR is the best-performing method for colon tumor staging for all the models. For liver tumor staging re-ranking methods improve the results for BioMedClip and SwinTransformer embeddings but show a decline for DreamSim embeddings, mirroring the trend observed in tumor flagging. For staging lung tumors, C-MIR shows the best outcomes for DreamSim embeddings but both re-ranking methods show declined performance for BioMedClip and SwinTransformer embeddings. In pancreas tumor staging, C-MIR is the best-performing method for BioMedClip and SwinTransformer embeddings, but the performance drops for DreamSim and SwinTransformer embeddings.

C-MIR achieved the best AP of 0.642 for colon tumor staging using DreamSim embeddings. For liver tumor staging, the C-MIR method using BioMedClip embeddings achieves the highest AP of 0.674. The best-performing method for lung tumor staging is C-MIR using DreamSim embeddings, with an AP of 0.588. In the staging of pancreatic tumors, C-MIR utilizing SwinTransformer embeddings achieves the highest AP of 0.660. In summary, C-MIR achieves the best performance for tumor staging across all four anatomical sites.

### Statistical Analysis

Sections“Tumor Flagging” and“Tumor Staging” showed that the C-MIR method exhibits varying performance levels when applied to different organs and datasets. Although C-MIR enhances tumor flagging and staging for specific organs and models, there are cases, especially with DreamSim embeddings, where the performance drops. These variations highlight the need for statistical analysis to evaluate the significance of the findings. To this end, we employed a two-sided Wilcoxon signed-rank test to assess the average precision of the C-MIR method against the best method for each database. The statistical test serves two purposes: First, it evaluates whether instances where C-MIR outperforms other methods reflect statistically significant improvements rather than random chance. Second, it assesses whether any observed declines in C-MIR’s performance, indicated by a lower average compared to other methods, are statistically significant. This approach aims to ensure that any changes in performance metrics are meaningful and reliable, rather than random variations.

#### Tumor Flagging

Table 7 contains the respective *p*-values for tumor flagging. The C-MIR method shows statistically significant improvements over the three vanilla aggregation methods and the RRF re-ranking in colon tumor flagging across all databases and models, highlighting its robustness in this application. For liver tumor flagging using BioMedClip and SwinTransformer embeddings, re-ranking methods do not show statistically significant improvements despite improvements in average APs. For the DreamSim model, re-ranking even declines the performance. C-MIR demonstrates statistically significant enhancements in lung tumor flagging for DreamSim embeddings when applied to organ-specific databases. However, the performance of C-MIR for the organ-agnostic database in combination with the BioMedClip embeddings shows a decline. C-MIR shows a subtle improvement in flagging pancreas tumors, only enhancing the results of BioMedClip embeddings in the organ-agnostic database, while its performance decreases for DreamSim embeddings in the organ-specific database without segmentation. The other differences in APs are not statistically significant.

**Table 7:** Wilcoxon test on average precision for **tumor flagging** of C-MIR versus the best-performing method

Organ	Model	Method	Organ-specific	Organ-specific	Method	Organ-agnostic
			Database w.	Database wo.		Database
			Segmentation	Segmentation		(*p*-value)
			(*p*-value)	(*p*-value)		
Colon	BioMedClip	Count-base	**.002**	**.002**	Count-base	**.004**
	DreamSim	RRF	**.003**	**.048**	RRF	**.001**
	SwinTrans.	RRF	**.001**	**.001**	RRF	**.001**
Liver	BioMedClip	RRF	.275	.322	RRF	.769
	DreamSim	Sum-Sim	**.002**	**.002**	Sum-Sim	**.002**
	SwinTrans.	RRF	.921	.160	Count-base	.275
Lung	BioMedClip	RRF	.431	.695	RRF	**.009**
	DreamSim	Count-base	**.037**	**.049**	Sum-Sim	.232
	SwinTrans.	RRF	**.083**	1.000	Count-base	.922
Pancreas	BioMedClip	Count-base	.232	.770	RRF	**.001**
	DreamSim	Count-base	.160	**.014**	Sum-Sim	.557
	SwinTrans.	Count-base	.492	.695	Sum-Sim	.557

The bold-faced values highlight the *p*-values smaller than 0.05. The underlined methods indicate where C-MIR, on average, performed worse than the specified method. In all other instances, C-MIR demonstrated improvements in average AP scores in Sections“Tumor Flagging” and“Tumor Staging”

**Table 8:** Wilcoxon test on average precision for **tumor staging** of C-MIR versus the best-performing method

Organ	Model	Method	Organ-specific	Organ-specific	Method	Organ-agnostic
			Database w.	Database wo.		Database
			Segmentation	Segmentation		(*p*-value)
			(*p*-value)	(*p*-value)		
Colon	BioMedClip	Count-base	.846	.432	Count-base	1.000
	DreamSim	RRF	.193	.232	RRF	**.001**
	SwinTrans.	RRF	**.001**	**.001**	RRF	**.005**
Liver	BioMedClip	Count-base	.322	.131	RRF	1.000
	DreamSim	Count-base	.105	**.027**	Sum-Sim	**.020**
	SwinTrans.	RRF	.625	.160	RRF	.625
Lung	BioMedClip	Count-base	.275	.131	Sum-Sim	**.049**
	DreamSim	Sum-Sim	.432	.193	Sum-Sim	.770
	SwinTrans.	Count-base	.375	.846	Count-base	.625
Pancreas	BioMedClip	Count-base	.232	.922	Count-base	**.002**
	DreamSim	Count-base	**.006**	**.004**	Sum-Sim	.846
	SwinTrans.	Count-base	.105	.557	Sum-Sim	.557

The bold-faced values highlight the *p*-values smaller than 0.05. The underlined methods indicate where C-MIR, on average, performed worse than the specified method. In all other instances, C-MIR demonstrated improvements in average AP scores in Sections“Tumor Flagging” and“Tumor Staging”

#### Tumor Staging

Table 8 presents *p*-values from the two-sided Wilcoxon signed-rank test comparing the average precision of the C-MIR method with the top-performing method for each database in tumor staging. In colon tumor staging, the C-MIR method demonstrates statistically significant enhancements using the DreamSim embeddings in organ-agnostic database and SwinTransformer embeddings across all databases. In liver tumor staging, a similar trend as flagging is noted: C-MIR reduces performance with DreamSim embeddings. For other models, although there was an increase in AP, these improvements are not statistically significant. Lung tumor staging is particularly difficult with no improvement in overall performance using C-MIR or RRF. The C-MIR method shows statistically significant improvements for pancreatic tumor staging, particularly with the DreamSim embedding and organ-specific database. For the organ-agnostic database, C-MIR shows statistically significant improvements only for the BioMedClip embeddings.

## Discussion

In this study, we conducted a comprehensive evaluation of CBIR systems for 3D medical image retrieval, with a particular emphasis on tumor flagging and staging. Our work builds upon existing methods, extending the evaluation to databases of varying configurations. We introduced the novel ColBERT-Inspired Medical Image Retrieval and Re-Ranking (C-MIR) method, which takes into account the information of the whole volume for re-ranking the top-k retrieved cases. We compared C-MIR with a meta re-ranking method and three vanilla retrieval methods that do not re-rank.

### Performance of C-MIR

Our findings demonstrate that C-MIR maintains consistent performance across databases, regardless of whether the images are retrieved from an image-only or a segmentation-mask-enhanced database (the latter being designed for precise organ-specific slice selection). This indicates that the additional 3D context information encoded in C-MIR’s similarity matrices improves localization of relevant anatomical regions without requiring prior localization, e.g., by segmentation. Since C-MIR only relies on slice embeddings that are needed for the vector similarity search anyway, this method is a computationally efficient alternative to search systems that rely on prior image segmentation or related types of computationally expensive data enrichment. This advantage is particularly evident when dealing with large volumetric image databases. In contrast, retrieval methods lacking 3D image context show performance variability (See Appendix 2 for a detailed statistical analysis). For these approaches, using an organ-specific database with pre-selected slices that exclude non-informative background, e.g., by organ segmentation (similar to the organ-specific database with segmentation), can improve tumor flagging, depending on the embedding or aggregation method. C-MIR provides a mean to eliminate this dependency, achieving equivalent performance while avoiding any kind of slices pre-filtering, e.g., by utilizing a segmentation model. This is a significant advantage in resource-constrained clinical settings where large-scale segmentation is often impractical.

We showed that the C-MIR method can be used effectively in the context of CBIR for medical image data in the presence of pathologies (here, tumors). Specifically, C-MIR could improve tumor flagging, in colon and lung cases. Given the correct choice of embedding C-MIR performs well for liver and pancreas tumor flagging as well (best or second-best). It is noteworthy that the effectiveness of this method as well as other methods varied depending on the embedding model, especially for larger databases. Conceptually, this is not a weakness of the C-MIR framework itself, as the embedding generation can easily be updated at any time to the latest available state-of-the-art models. In other words, as increasingly more foundation models with the capability to generalize on broader tasks become available in the future, medical image retrieval will also become more accurate. For tumor staging, the results were more variable, suggesting that further refinement of these methods is necessary to improve performance. C-MIR had the highest APs for tumor staging for all the organs in comparison with vanilla aggregation methods in the organ-agnostic database. Nevertheless, the results revealed areas where the method did not achieve any significant improvements, indicating a need for further research.

### Challenges in Tumor Staging

Automated tumor staging faces significant challenges due to the clinical staging requirements and workflows. Tumor staging relies on precise, scale-dependent features such as absolute physical size (e.g., tumor diameter in millimeters) and anatomical context, which clinicians derive from raw medical images and image metadata like pixel spacing and slice thickness. This aspect of CBIR systems warrants additional research to ensure that critical image details are preserved and accurately represented in the retrieval process. In contrast, tumor flagging generally yields better results since it primarily focuses on the presence of tumors rather than their size and other detailed characteristics. When moving from flagging to staging, the importance of these detailed characteristics becomes increasingly significant, as staging requires a more nuanced analysis that takes into account the exact dimensions and growth patterns of the tumor. Hence, while flagging can be effectively handled by the current CBIR approach, staging necessitates advancements in preserving and utilizing the full range of image details to improve retrieval accuracy. To enhance the effectiveness of tumor staging, future studies should focus on utilizing higher-resolution images and fine-grained details, using multi-resolution approaches or leveraging anatomical landmarks (e.g., vertebrae, blood vessels) as intrinsic reference points to estimate tumor size proportionally.

### Limitations and Potentials of the C-MIR Method

Furthermore, it is crucial to acknowledge the limitations of re-ranking methods. Since re-ranking only modifies the order of the top retrieved cases, its effectiveness is inherently dependent on the initial retrieval quality. If the first retrieval does not return relevant cases among the top results, the effectiveness of re-ranking solutions, including C-MIR, is limited. This highlights the importance of robust initial retrieval mechanisms to fully leverage the benefits of re-ranking methods such as C-MIR. While we utilized C-MIR for re-ranking in this study, it is worth noting that the C-MIR approach, with its full embedding matrix, could also be applied as a primary retrieval system. However, such an application would require loading the matrices of volumes into memory, which is feasible only for small datasets due to the substantial computational resources it demands. Future research can focus on exploring the scalability of C-MIR and its application to larger datasets for image retrieval, as well as enhancing the initial retrieval mechanisms to improve overall re-ranking performance.

### Scalability and Computational Efficiency

C-MIR is used as a re-ranking method here to ensure scalability for large datasets. Based on an initial top-k similarity search, C-MIR is applied only to these top-k candidate volumes. It only relies on the vector embeddings related to the slices of the query and the top-k image volumes. This significantly reduces the computational burden. For example, with an embedding dimension of 1024, a query volume of 300 slices, and re-ranking the top 20 candidate volumes (each with 250–500 slices), matrix multiplications overall require approximately 6.14B FLOPs (307.2M FLOPs per each matrix multiplication) and $<15$MB of GPU memory (assuming 32-bit floating point precision). Modern GPUs and CPUs can easily handle this workload in milliseconds, and the small memory footprint allows for efficient processing.

The re-ranking approach ensures that the computational cost scales primarily with the number of top-k candidates considered for re-ranking, not the overall database size. Furthermore, the computation for each candidate volume is independent, allowing for efficient parallelization via batch processing. This makes C-MIR a scalable solution for improving retrieval accuracy in large-scale datasets, maintaining robust performance even as the dataset grows, while remaining computationally tractable.

### Future Directions for Re-ranking Methods

Most existing re-ranking approaches in the literature are developed for text retrieval or 2D image domains. Future work could focus on adapting these methods to handle the unique challenges of volumetric data, particularly the inherent variability in slice counts across medical volumes. Such adaptations would need to address computational efficiency and memory constraints inherent to 3D data. Comparative evaluation of these adapted methods against C-MIR would help identify optimal strategies for volumetric re-ranking, particularly in scenarios requiring fine-grained similarity assessment across variable-length volumes. This exploration could also reveal whether techniques successful in text/2D domains (e.g., late interaction, cross-attention mechanisms) generalize effectively to 3D medical imaging.

Furthermore, a critical direction for future research is the validation of these re-ranking methods on independent, external datasets. This is essential to assess their generalizability to real-world clinical data and to ensure that the observed performance gains are not specific to the public dataset used in this study. Such external validation should ideally involve datasets from multiple institutions with varying imaging protocols and patient populations to provide a robust assessment of the methods’ clinical utility.

## Conclusion

In this study, we introduced a novel re-ranking and retrieval approach called C-MIR, inspired by the principles of ColBERT, where 2D slices (analogous to words) and 3D volumes (analogous to passages) are encoded into multi-vector representations using pre-trained vision models. By computing maximum cosine similarities between query slices and all slices in retrieved volumes, C-MIR leverages the inherent three-dimensional spatial context of radiological data to refine relevance rankings. We showed that C-MIR can be used in the context of CBIR retrieval and improve the outcome, especially in tumor flagging. Additionally, our evaluation demonstrates that C-MIR can effectively localize regions of interest by incorporating context similarity. The proposed method demonstrates computational efficiency and scalability for large, unannotated datasets, offering practical value for real-world clinical applications. While the method reliably flags tumor presence in retrieved cases−a critical first step for diagnostic workflows−the tumor stage of retrieved instances showed variability across experiments. This indicates that while C-MIR effectively identifies tumor-afflicted cases, refining its ability to match precise staging criteria remains an important focus for future work. This study establishes a basis for future research to create more robust and efficient retrieval techniques by leveraging an existing method without requiring prior segmentation or organ-specific databases. Our findings contribute to the growing body of literature on CBIR in the medical domain, emphasizing the urgent need for reliable and efficient retrieval methods that can be seamlessly integrated into clinical workflows.

## Figures and Tables

**Fig. 5:**
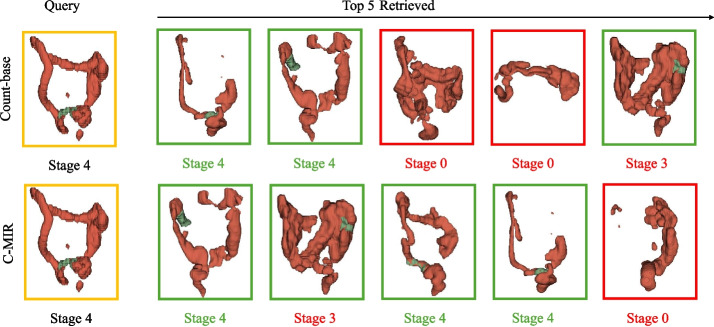
Visual representation of retrieval outcomes for one colon case, with the top five retrieved results. The colon segmentation mask is shown in red, and the tumors are denoted in green. Green boxes indicate accurate tumor flags, while red boxes indicate failures. Stage numbers below each instance show the actual and matched stages, with green for correct and red for incorrect matches. C-MIR improved tumor flagging and tumor staging for the top five retrieved cases

**Fig. 6:**
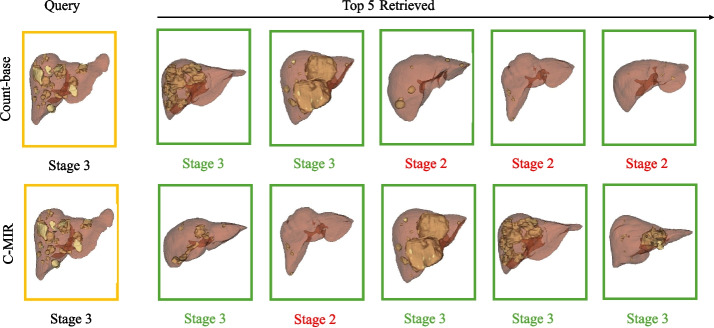
Visual representation of retrieval outcomes for one liver case, with the top five retrieved results. The liver segmentation mask is shown in brown, and the tumors are denoted in yellow. Green boxes indicate accurate tumor flags, while red boxes indicate failures. Stage numbers below each instance show the actual and matched stages, with green for correct and red for incorrect matches. C-MIR improved tumor staging for the top five retrieved cases. Both count-base and C-MIR methods indicate a perfect tumor flagging score for the top five retrieved cases

**Fig. 7:**
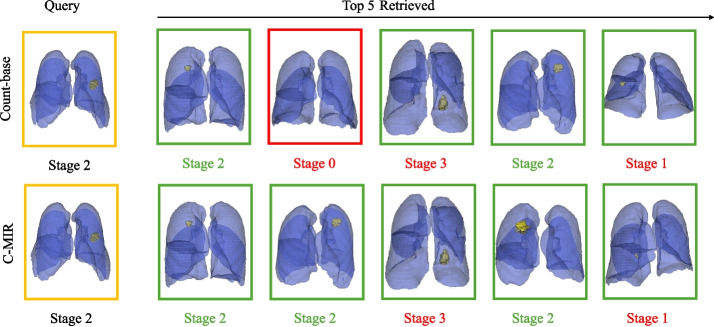
Visual representation of retrieval outcomes for one lung case, with the top five retrieved results. The lung segmentation mask is shown in blue, and the tumors are denoted in yellow. Green boxes indicate accurate tumor flags, while red boxes indicate failures. Stage numbers below each instance show the actual and matched stages, with green for correct and red for incorrect matches. C-MIR improved tumor flagging and staging for the top five retrieved cases

**Fig. 8:**
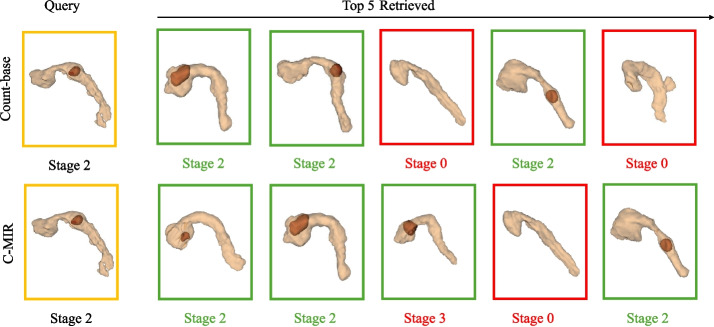
Visual representation of retrieval outcomes for one pancreas case, with the top five retrieved results. The pancreas segmentation mask is shown in yellow, and the tumors are denoted in brown. Green boxes indicate accurate tumor flag, while red boxes indicate failures. Stage numbers below each instance show the actual and matched stages, with green for correct and red for incorrect matches. C-MIR improved tumor flagging; however, the tumor staging score remains unchanged for the top five retrieved cases

## Data Availability

The details of model versions and data splits, including query and database sets, are available upon request. Interested parties can contact the corresponding author for further information on accessing the data.

## Appendix 1: Qualitative examples

Figures. 5, 6, 7, and 8 visually depict the top five retrieval outcomes for the colon, liver, lung, and pancreas tumors, utilizing embeddings from the SwinTransformer. The figures compare two retrieval approaches: count-base and C-MIR. The chosen cases illustrate scenarios where C-MIR either enhanced tumor flagging or staging or improved both aspects. TotalSegmentator model [24] is used for organ segmentation, while tumor segmentations are obtained from MSD tumor masks [23]. It is worth mentioning that some organ segmentations are incomplete due to the automatic segmentation process. In every figure, the query serves as the input image for the search system, and the top five retrieved results are displayed in the same row. The green boxes indicate instances where the tumor flagging was accurate, whereas the red boxes represent instances where tumor flagging was unsuccessful. Below each query or retrieved instance, there is a stage number provided. The stage number shows the actual stage of the query and the corresponding stages of the matched cases. The color of the stages indicates whether the tumor stage is matched correctly, with green for a correct match and red for an incorrect match. The colors of the boxes and stages are independent. For instance, a tumor can be flagged without the correct stage classification, which is indicated by green boxes and red text. It should be noted that the cases presented here were selected from the test set to illustrate common success/failure modes. Full quantitative metrics are reported in Section“Results and Evaluation.”

Figure 5 illustrates a query containing a colon with a stage 4 tumor. The first row shows the top five cases retrieved using the count-base method, with three cases exhibiting a tumor in stages 4, 4, and 3, respectively. Here, P@3 and P@5 for tumor flagging are $66\%$ and $60\%$, and P@3 and P@5 for tumor staging are $66\%$ and $40\%$. The second row shows the top five cases retrieved using the C-MIR method as re-ranker, with four cases containing a tumor in stages 4, 3, 4, and 4, respectively. Here, P@3 and P@5 for tumor flagging are $100\%$ and $80\%$, and P@3 and P@5 for tumor staging are $66\%$ and $60\%$. This case demonstrates an example where the C-MIR re-ranking improves both tumor flagging and tumor staging.

Figure 6 depicts a query containing a liver with multiple tumors, classified as a stage 3 case according to the count and size of the tumors. The first row displays the top five cases retrieved by the count-base method, all of which have tumors. However, only two of the top retrieved cases contain stage 3 cases, and the rest are stage 2. Thus, P@3 and P@5 for tumor flagging are $100\%$, and P@3 and P@5 for tumor staging are $66\%$ and $40\%$. The second row shows the top five retrieved cases using the C-MIR method as re-ranker, again with all cases containing tumors. Here, four out of five cases contain tumors of stage 3. As a result, P@3 and P@5 for tumor flagging are $100\%$, and P@3 and P@5 for tumor staging are $66\%$ and $80\%$. This case demonstrates an example where the C-MIR re-ranking improves tumor staging for the top five cases.

Figure 7 demonstrates a query containing a lung with a stage 2 tumor. The first row displays the top five cases retrieved by the count-base method. Four out of five retrieved cases contain tumors of stages 2, 3, 2, and 1. Thus, P@3 and P@5 for tumor flagging are $66\%$ and $80\%$, and P@3 and P@5 for tumor staging are $33\%$ and $40\%$. The second row shows the top five retrieved cases using the C-MIR method as re-ranker with all cases containing a tumor. Here, three out of five cases contain tumors of stage 2. Therefore, P@3 and P@5 for tumor flagging are $100\%$, and P@3 and P@5 for tumor staging are $66\%$ and $60\%$. In this case, C-MIR re-ranking improved both tumor flagging and tumor staging for the top five cases.

Figure 8 depicts a query containing a pancreas with a stage 2 tumor. The first row displays the top five cases retrieved by the count-base method where three out of five retrieved cases contain tumors of stage 2. Thus, P@3 and P@5 for both tumor flagging and staging are $66\%$ and $60\%$. The second row shows the top five retrieved cases using the C-MIR method as re-ranker, with four out of five cases containing tumors of stages 2, 2, 3, and 2. Therefore, P@3 and P@5 for tumor flagging are $100\%$ and $80\%$, and P@3 and P@5 for tumor staging are $66\%$ and $60\%$. In this case, C-MIR re-ranking improved tumor flagging, but the tumor staging score remains the same.

## Appendix 2: Statistical analysis: retrieval results for organ-specific database with/without segmentation

Table 9 demonstrates the Wilcoxon test on average precision of tumor flagging and tumor staging using the organ-specific database with segmentation versus the organ-specific database without segmentation. The intention of the test is to show whether limiting the search space to organs has a statistically significant impact on the retrieval results for the vanilla aggregation approaches. Incorporating segmentation in creating databases significantly influences tumor flagging and staging outcomes, though its impact varies with the choice of image embedding and aggregation method. For tumor flagging, using the organ-specific database with segmentation frequently yielded statistically significant improvements ($p < 0.05$) across multiple organs, particularly when using the BioMedClip embedding (e.g., colon: $p=[0.037-0.049]$ for all aggregation methods; liver: $p=0.020$ for count-base/Sum-Sim). SwinTransformer embeddings also showed significant benefits from using pre-selected slices for flagging of colon and liver tumors ($p=[0.010-0.037]$). In contrast, DreamSim embeddings demonstrated more limited instances of significant improvement in flagging tasks with a similar setup. For tumor staging, the statistical significance of pre-selecting slices via segmentation of database cases was more specific. Notably, the SwinTransformer embeddings showed a highly significant improvement for colon tumor staging across all aggregation methods ($p=0.010$) and for lung tumor staging ($p=0.014$ for count-based/Sum-Sim). BioMedClip embeddings also showed selective benefits with pre-selected slices (e.g., lung Max-Score: *p* = 0.027; pancreas Max-Score: $p=0.010$). However, using pre-selcted slices for the database via segmentation did not yield statistically significant improvements for staging when using the DreamSim embeddings for any tested organ or aggregation method. These findings underscore that the benefit of the organ-specific database with segmentation is context-dependent, necessitating careful consideration of the embedding model and downstream task.

**Table 9:** Wilcoxon test on average precision of tumor flagging and tumor staging using organ-specific database with segmentation versus organ-specific database without segmentation

Organ	Model	Flagging *p*-values w/wo segmentation			Staging *p*-values w/wo segmentation		
		Count-base	Max-Score	Sum-Sim	Count-base	Max-Sim	Sum-Sim
sideways	BioMedClip	**0**.**049**	**0**.**037**	**0**.**037**	0.846	0.770	0.432
	DreamSim	0.375	**0**.**002**	0.322	0.375	0.084	0.131
	SwinTrans	**0**.**027**	0.625	**0**.**037**	**0**.**010**	**0**.**010**	**0**.**010**
Liver	BioMedClip	**0**.**020**	0.695	**0**.**020**	0.160	0.131	**0**.**037**
	DreamSim	0.557	**0**.**040**	0.375	0.770	0.922	0.695
	SwinTrans	**0**.**027**	0.846	**0**.**010**	0.160	0.084	0.064
Lung	BioMedClip	0.105	**0**.**010**	0.275	0.275	**0**.**027**	0.432
	DreamSim	0.695	0.109	0.625	0.160	0.093	0.441
	SwinTrans	0.625	**0**.**036**	0.695	**0**.**014**	0.432	**0**.**014**
Pancreas	BioMedClip	0.064	**0**.**027**	**0**.**010**	0.131	**0**.**010**	0.064
	DreamSim	0.064	1.000	**0**.**037**	0.232	0.432	0.084
	SwinTrans	0.375	0.160	0.275	0.160	0.625	0.084

The bold-faced values highlight the *p*-values smaller than 0.05
